# Challenges and Opportunities of Direct Oral Anticoagulant (DOAC) Therapy in Complex Clinical Scenarios: A Comprehensive Review and Practical Guide

**DOI:** 10.3390/jcm14092914

**Published:** 2025-04-23

**Authors:** Giuseppe Miceli, Anna Maria Ciaccio, Antonino Tuttolomondo

**Affiliations:** 1 Department of Health Promotion, Mother and Child Care, Internal Medicine and Medical Specialties (ProMISE) Università degli Studi di Palermo, Piazza delle Cliniche 2, 90127 Palermo, Italy; 2 Internal Medicine and Stroke Care Ward, University Hospital, Policlinico “P. Giaccone”, 90100 Palermo, Italy

**Keywords:** direct oral anticoagulants, thrombosis, anticoagulation

## Abstract

Direct oral anticoagulants (DOACs) have emerged as a preferred alternative to vitamin K antagonists (VKAs) for the prevention and treatment of thromboembolic disorders, offering improved safety, predictable pharmacokinetics, and ease of administration. Despite these advantages, their use in complex clinical scenarios presents significant challenges that necessitate individualized therapeutic strategies. This comprehensive review explores the efficacy, safety, and limitations of DOAC therapy in special populations, including patients with renal or hepatic impairment, obesity, cancer-associated thrombosis, and antiphospholipid syndrome. Additionally, we examine their role in uncommon thrombotic conditions such as superficial venous thrombosis, embolic stroke of undetermined source, upper extremity vein thrombosis, inferior vena cava thrombosis, pelvic vein thrombosis, and cerebral vein thrombosis. The pharmacokinetic variability of DOACs in renal and hepatic dysfunction requires caution to balance the bleeding and thrombotic risks. In obesity, altered drug distribution and metabolism raise concerns regarding appropriate dosing and therapeutic efficacy. Cancer-associated thrombosis presents a complex interplay of prothrombotic mechanisms, necessitating careful selection of anticoagulant therapy. Furthermore, the use of DOACs in antiphospholipid syndrome remains controversial due to concerns about recurrent thrombotic events. Finally, in some unusual scenarios like inferior vena cava, pelvic vein, and cerebral vein thrombosis, the use of DOACs has scarce evidence. This review aims to guide clinicians in optimizing anticoagulation management in challenging patient populations by synthesizing current evidence and providing practical recommendations.

## 1. Introduction

Direct oral anticoagulants (DOACs) are a group of agents used to prevent thrombosis in several clinical conditions [1]. They have quickly become reliable alternatives to the long-standing standard of care in anticoagulation, the vitamin K antagonist (VKAs).

For more than 60 years, thromboembolic events were primarily treated with VKAs, such as warfarin, which acts by reducing the synthesis of functional vitamin K-dependent factors, including FII, FVII, FIX, FX, protein C, and protein S, by interfering with the vitamin K redox cycle. While effective, the use of these agents is challenging due to dietary restrictions, frequent monitoring by measuring international normalized ratio (INR), and complex dosing regimens. Indeed, maintaining a stable and therapeutic INR can be difficult due to interactions with drugs, food, and liver disease, resulting in an increased risk of thromboembolism from undertreatment or bleeding from overtreatment. Thus, the pharmacokinetics and pharmacodynamics of VKAs are unpredictable. Additionally, VKAs have a slow onset, taking 2–7 days to be effective. Consequently, VKA therapy is initially co-administered with other anticoagulants, such as heparin [2]. Finally, long-term administration of VKAs can result in side effects, such as vascular calcification, anticoagulation-related nephropathy, and osteoporosis [3].

DOACs were developed to overcome the drawbacks of VKA therapy while maintaining safety profiles.

Regulatory agencies have approved several DOACs based on clinical trials showing that they are at least as effective and safe as VKAs or low-molecular-weight heparin (LMWH). Overall, DOACs offer several advantages over VKAs.

In the general population, DOACs revolutionized the prevention as well as treatment of venous thromboembolism (VTE) and primary and secondary prevention of ischemic stroke and systemic embolism in patients with atrial fibrillation (AF). However, their use in specific clinical scenarios presents unique challenges. Certain patient populations, such as those with renal or hepatic impairment, obesity, cancer-associated thrombosis, or antiphospholipid syndrome, require careful consideration due to altered pharmacokinetics, potential drug interactions, and variable efficacy or safety outcomes.

This article provides an in-depth overview of the literature on the safety and efficacy of DOACs in these clinical conditions, highlighting their advantages, limitations, and potential areas for future research. By addressing the nuances of DOAC therapy in special populations, this article seeks to provide clinicians with practical guidance for optimizing anticoagulation management in complex clinical settings.

## 2. DOACs Characteristics

**Figure 1:**
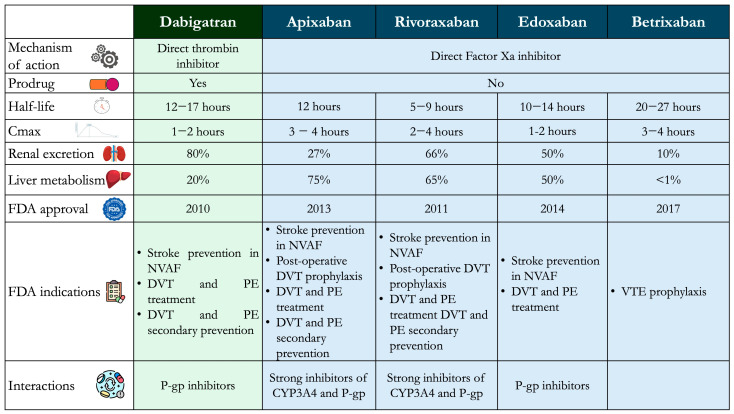
DOACs characteristics. NVAF, non-ventricular atrial fibrillation; P-gp, P-glycoprotein; DVT, deep vein thrombosis; PE, pulmonary embolism.

In 2010, the US Food and Drug Administration (FDA) approved the first DOAC, dabigatran, followed by rivaroxaban, apixaban, edoxaban, and betrixaban in subsequent years. DOACs directly inhibit an activated clotting factor. Based on their target, they can be categorized into two classes: (i) FXa inhibitors, including rivaroxaban, apixaban, edoxaban, and betrixaban; (ii) direct thrombin inhibitors, including dabigatran. Figure 1 summarizes the main characteristics of DOACs. Over the years, several terms have been proposed for DOACs, including novel/new oral anticoagulants (NOACs) and target-specific oral anticoagulants (TOACs). In 2015, the Control of Anticoagulation Scientific and Standardization Committee of the International Society on Thrombosis and Haemostasis (ISTH) proposed universally using the term DOAC [4].

They have predictable pharmacokinetics and pharmacodynamics, eliminating the need for routine INR monitoring and reducing dietary and drug interactions, and they are administered orally. These advantages have led to the widespread adoption of DOACs across various indications in different clinical conditions and their implementation into daily care.

## 3. DOACs in Different Clinical Scenarios

### 3.1. DOACs in Chronic Liver Diseases

**Figure 2:**
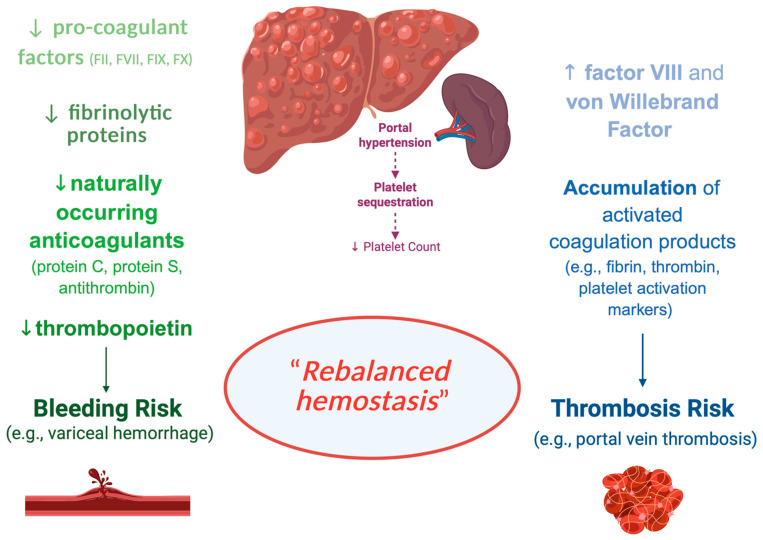
Complex hemostatic alterations in chronic liver diseases.

The liver plays a critical role in the hemostatic system by synthesizing most coagulation factors, molecules involved in fibrinolysis, and thrombopoietin, the key hormone for platelet production from megakaryocytes. Thus, chronic liver diseases (CLDs) influence the hemostatic system [5] (Figure 2).

Specifically, patients with CLD have decreased levels of hemostatic factors, which are balanced by the increase in hemostatic factors, such as von Willebrand factor (VWF), produced by endothelial cells, which are under a continuous low-grade activation status. In addition, portal hypertension, the major complication of CLD, results in splenomegaly, leading to increased platelet sequestration in the spleen and contributing to thrombocytopenia in CLD [6]. In summary, the hemostatic profile of a patient with CLD typically includes thrombocytopenia, reduced levels of hemostatic factors and inhibitors, reduced levels of fibrinolytic proteins, and increased plasma levels of FVIII and VWF. Hemostasis is a dynamic interplay among platelets, coagulation factors, inhibitors, and fibrinolytic proteins. In patients with CLDs, extremely complex alterations of hemostasis occur. Although CLDs were historically regarded as bleeding disorders, it is now recognized that they are associated with both bleeding and thrombotic complications.

Clinical studies revealed an increased risk of VTE, pulmonary embolism, stroke, and myocardial infarction in patients with CLD [7,8,9].

Increased levels of platelet activation markers, thrombin, fibrin production, and fibrinolysis are common in CLD patients. However, since these factors are cleared by the liver, their increased levels could result from defective clearance rather than ongoing activation of platelets, coagulation, and fibrinolysis [5].

Rebalanced hemostasis has become widely embraced due to concurrent changes in both pro- and anti-hemostatic processes [10]. However, the rebalanced hemostatic status in CLDs is not as stable as in healthy individuals and can be disturbed by disease complications, such as infections and renal failure. Consequently, although the hemostatic balance is maintained, even in patients with end-stage cirrhosis, it may shift towards either a hypo- or hypercoagulable state in individual patients, explaining the occurrence of both bleeding and thrombotic episodes.

Due to the lack of high-quality clinical evidence, it is still unclear how to prevent or treat bleeding and thrombosis in CLDs. Notably, cirrhosis may be complicated by portal vein thrombosis (PVT), which is rare in the general population but common in CLD patients. Thus, CLD patients may benefit from anticoagulant therapy. However, they were excluded from the approved clinical trials of DOACs for VTE treatment due to their complex hemostatic status and altered pharmacokinetics. This resulted in a clinically important knowledge gap on the efficacy and safety of DOACs in patients with CLD.

While warfarin is primarily metabolized by cytochrome enzymes and relies on the liver for clearance, DOACs are generally less reliant on these enzymes for metabolism. They are metabolized in the liver to varying extents (75% apixaban, 65% rivaroxaban, 50% edoxaban, and 20% dabigatran).

Lawal et al. performed a large retrospective cohort study in adults with acute VTE and CLD, including cirrhosis, metabolic dysfunction-associated fatty liver disease (MAFLD), and viral hepatitis [11]. The cohorts consisted of 2.361 DOAC-warfarin, 895 apixaban-warfarin, 2.161 rivaroxaban-warfarin, and 895 apixaban-rivaroxaban matched pairs. The authors showed that DOAC therapy was associated with significantly lower risks of hospitalization for major bleeding and recurrent VTE than warfarin therapy. No difference was observed in the risk of all-cause mortality for exposure to any or specific DOACs vs. warfarin. Thus, DOACs have a more favorable benefit–risk profile than warfarin. DOACs may serve as an alternative to warfarin in individuals with VTE and cirrhosis. This study provides the most robust real-world evidence to date that DOACs are effective and safe in patients with CLDs.

The same research group performed a large retrospective study, including 10.209 patients with CLD and atrial fibrillation who were new users of anticoagulant therapy with DOACs, apixaban, rivaroxaban, or warfarin [11]. The authors showed that patients treated with DOACs had a 36% lower risk for ischemic stroke, a 24% lower risk of all-cause death, a 31% lower risk of hospitalization for major bleeding, and a 27% lower risk of hospitalization for major gastrointestinal bleeding. In a head-to-head DOAC comparison, the risks for effectiveness outcomes, i.e., ischemic stroke and mortality, were not significantly different between rivaroxaban and apixaban. However, the risk for major bleeding was markedly higher for rivaroxaban versus apixaban. Thus, the study provides preliminary evidence suggesting that DOACs may be effective and safe in patients with CLD and AF, though further prospective studies are warranted to confirm these findings.

These findings are in accordance with previous small retrospective studies suggesting that DOACs are a safe treatment option in patients with CLD [12].

Attention must be paid to patients with cirrhosis. The European Association for the Study of the Liver (EASL) and the European Heart Rhythm Association recommend against the use of DOACs in patients with Child–Pugh C cirrhosis due to the increased incidence of variceal hemorrhage and low one-year survival rates without liver transplantation, making the avoidance of oral anticoagulation in this group reasonable [13]. The American Gastroenterology Association suggests anticoagulation in cirrhotic patients with AF when indicated, but advises that patients with Child–Pugh C cirrhosis or a low CHA2DS2-VA score might opt out of anticoagulation [14]. The EASL indicates no formal contraindications for DOAC use in patients with Child–Pugh A cirrhosis [15]. Contrasting evidence regards the use of DOACs in patients with Child–Pugh B cirrhosis. The European Association for the Study of the Liver advises that DOACs should be used cautiously in these patients [13], while the European Heart Rhythm Association recommends avoiding DOACs in patients with Child–Pugh B cirrhosis entirely [13]. Thus, all DOACs can be used in patients with mild hepatic dysfunction (Child–Pugh A) with no bleeding risk [13]. In patients with moderate hepatic dysfunction (Child–Pugh B), dabigatran, apixaban, and edoxaban can be used with caution, while rivaroxaban should not be used because of increased plasma concentrations and pharmacodynamic effects [16]. In severe hepatic dysfunction (Child-Pugh C), DOACs are not recommended. Thus, DOACs should be used with caution in patients with advanced chronic liver disease due to a significant rate of spontaneous bleeding events.

As PVT is a common complication of advanced liver cirrhosis, associated with a worse prognosis, some Authors evaluated the safety and effectiveness of DOACs for treating PVT in cirrhotic patients. PVT is defined as the presence of a thrombus within the portal vein, which can extend to the splenic or superior mesenteric vein. According to the degree of obstruction, it can be classified as occlusive or nonocclusive. Although anticoagulant therapy is the pillar treatment for acute symptomatic PVT, the effectiveness of anticoagulant therapy in chronic PVT is less clear [17]. The literature evidence suggests that anticoagulant therapy in cirrhotic patients with PVT is safe and effective, resulting in portal vein recanalization, reduced PVT progression, and increased survival rate [18]. Notably, rivaroxaban, apixaban, and edoxaban act independently of endogenous antithrombin. This could be advantageous in cirrhotic patients in whom antithrombin is reduced. However, evidence on the efficacy of DOACs to treat PVT in cirrhosis is limited [19,20]. The available data suggest that DOACs are noninferior compared with VKAs therapy in terms of efficacy, while the safety of DOACs seems superior [21]. However, the studies have important limitations, such as the small sample size and the heterogeneity of criteria for treatment duration or dosage strategy. Thus, randomized controlled trials are mandatory to assess the safety and efficacy of DOACs in treating PVT in cirrhotic patients.

Douros et al. assessed the effectiveness and safety of DOACs in patients with non-valvular atrial fibrillation (NVAF) and liver disease (LD), including both acute and chronic conditions [22]; fatty liver (48%), followed by cirrhosis (23%), were the most common LD. The HR (95% CI) for DOACs compared to VKAs was 1.01 (0.76 to 1.34) (I2 = 0%) for ischemic stroke and 0.87 (0.77 to 0.99) (I2 = 48%) for major bleeding. The HR (95% CI) for all-cause mortality was 0.90 (0.81 to 1.01) (I2 = 46%). Overall, the authors did not find major differences in effectiveness between DOACs and warfarin, but a small safety benefit was observed with DOACs among patients with NVAF and liver disease. Interestingly, the findings did not change when focusing on patients with cirrhosis, arguing that even advanced liver disease does not alter the benefit–risk balance between DOACs and warfarin.

Similarly, Lee et al., in a large cohort study from South Korea, showed the strong beneficial effects of DOACs in patients with NVAF and liver disease [23]. Compared to warfarin, DOACs were associated with a 36% decreased risk for ischemic stroke, 31% for major bleeding, and 24% for all-cause mortality.

The use of DOACs has also been explored in patients with Budd–Chiari Syndrome (BCS), a rare disorder due to the obstruction of hepatic venous outflow, which may occur anywhere from the small hepatic veins to the junction of the inferior vena cava (IVC) and the right atrium [24]. The primary cause of BCS is thrombosis of the hepatic veins. Predisposing conditions often include myeloproliferative neoplasms, such as polycythemia vera, inherited thrombophilias, paroxysmal nocturnal hemoglobinuria, and autoimmune diseases. The treatment is guided by disease severity and the underlying cause, with anticoagulation being the cornerstone of therapy. Traditionally, vitamin K antagonists (VKAs) like warfarin have been used for long-term anticoagulation in BCS. However, direct oral anticoagulants (DOACs) are increasingly being used. Recent observational studies and case series have demonstrated that DOACs are safe and effective alternatives to VKAs in BCS, especially in stable, non-cirrhotic patients [25]. While the literature supports the use of DOACs in BCS, several limitations must be acknowledged. The studies and case reports are all retrospective, which inherently introduces potential biases and limits the strength of the conclusions that can be drawn. Additionally, due to the rarity of BCS, most studies included relatively small patient populations, further affecting the generalizability of the findings. The heterogeneity in BCS etiology, variability in underlying liver function, and differences in interventional approaches across studies also present significant challenges in standardizing outcomes and treatment protocols.

To better define the role of DOACs in BCS, future research should focus on large-scale, prospective, randomized controlled trials.

A significant number of patients with advanced CLD (ACLD) require anticoagulant therapy to prevent or treat venous thromboembolism and PVT. While DOACs appear to be safe for patients with compensated ACLD, there are limited data available for those with decompensated ACLD.

In clinical practice, a significant challenge is accurately assessing the hemostatic profile of patients with cirrhosis. Traditionally, these patients have been perceived as being at a heightened risk for bleeding complications. However, emerging evidence indicates that cirrhotic patients are also susceptible to thrombotic events, such as deep vein thrombosis and pulmonary embolism [26]. This dual risk necessitates a nuanced understanding of their coagulation status [27].

A prevalent source of bleeding in cirrhotic patients is variceal hemorrhage, primarily resulting from the rupture of esophageal or gastric varices [28]. The development and rupture of these varices are predominantly driven by hemodynamic alterations, notably increased pressure within the portal and splanchnic circulation, rather than intrinsic coagulation abnormalities. This distinction underscores that variceal bleeding is more a consequence of portal hypertension than a reflection of a systemic bleeding tendency.

The management of anticoagulation in cirrhotic patients presents a complex clinical dilemma. While anticoagulant therapy is essential for preventing thrombotic events, its use in patients with existing varices raises concerns. Specifically, if a patient on anticoagulation therapy experiences variceal rupture, the resultant hemorrhage can be more severe, and achieving hemostasis endoscopically may become more challenging. Therefore, it is imperative to carefully evaluate the risks and benefits of anticoagulation in this population. Prophylactic measures, such as routine endoscopic surveillance and the use of non-selective beta-blockers, may be considered to reduce the risk of variceal bleeding before initiating anticoagulant therapy [29,30].

Finally, reduced-dose DOACs have been explored as a potential strategy to mitigate bleeding risk while preserving efficacy, particularly in patients at higher risk or with moderate hepatic impairment (Child–Pugh A or B). A small number of retrospective studies and subgroup analyses have shown that dose-reduced regimens may still offer adequate thromboembolic protection in CLD without significantly increasing bleeding complications. However, due to the heterogeneity of liver disease and the paucity of randomized data, the decision to use reduced-dose DOACs should be individualized, ideally guided by hepatic function, bleeding risk, and clinical context, with close monitoring.

In conclusion, the hemostatic management of cirrhotic patients requires a balanced approach that considers both their thrombotic and hemorrhagic risks (Table 1). A comprehensive understanding of the underlying hemodynamic processes and vigilant monitoring can aid in optimizing therapeutic strategies for this complex patient population.

### 3.2. DOACs in Advanced-Stage Chronic Kidney Disease

Chronic kidney disease (CKD) is defined as kidney damage or glomerular filtration rate (GFR) < 60 mL/min/1.73 m^2^ for 3 months or more, irrespective of cause, according to the Kidney Disease Improving Global Outcomes (KDIGO) guidelines [31]. The KDIGO guidelines stratify CKD into five stages based on GFR and albuminuria, which reflect the severity of kidney damage and the risk of progression to end-stage renal disease (ESRD). CKD represents a global public health problem affecting over 10% of the general population [32]. AF is the most common arrhythmia worldwide [33]. Notably, the prevalence of CKD and AF grows with age. Up to 20% of patients have the co-occurrence of CKD and AF, resulting in an increased risk of stroke, cardiovascular morbidity, and all-cause mortality compared with patients with either AF or CKD alone [34]. Since DOACs, at a different rate, are at least partly renal-cleared (Table 1), their use in CKD patients requires careful consideration due to altered drug clearance and the potential for increased bleeding risk. Notably, patients with advanced-stage CKD have been excluded from phase 3 clinical trials. Thus, the efficacy and safety of DOACs in patients with advanced CKD, especially those receiving maintenance dialysis and AF, are less clear.

Randomized controlled trials (RCTs) have been performed to evaluate the safety and efficacy of DOACs compared to VKAs in patients with moderate CKD, defined as a creatine clearance (CrCl) ≥ 30–50 mL/min. The Rivaroxaban Once Daily Oral Direct Factor Xa Inhibition Compared with Vitamin K Antagonism for Prevention of Stroke and Embolism Trial in Atrial Fibrillation (ROCKET AF) was a multicenter, randomized, double-blind, double-dummy, event-driven trial that was conducted at 1178 participating sites in 45 countries [35]. Patients with atrial fibrillation were randomly assigned to receive fixed-dose rivaroxaban or adjusted-dose warfarin to prevent stroke and systemic embolism. In 21% of the cohort with moderate CKD, rivaroxaban was not inferior to warfarin in efficacy and safety. Similarly, the distinct trial J-ROCKET AF showed that rivaroxaban was not inferior to warfarin in terms of efficacy and safety in 284 patients with moderate kidney dysfunction and non-valvular AF at increased risk for stroke [36]. In the Apixaban for Reduction in Stroke and Other Thromboembolic Events in Atrial Fibrillation (ARISTOTLE) trial, apixaban was compared with warfarin for the prevention of stroke or systemic embolism in patients with atrial fibrillation and at least one additional risk factor for stroke [37]. Fifteen percent of the entire cohort had moderate kidney dysfunction. Apixaban was not inferior to warfarin in terms of efficacy but was superior in terms of safety, defined as a decreased risk of major bleeding. In the Randomized Evaluation of Long-Term Anticoagulation Therapy (RE-LY), the efficacy and safety of dabigatran were compared with warfarin in a cohort of 18,113 patients with atrial fibrillation at risk of stroke [38]. Among 3471 patients with moderate kidney dysfunction, dabigatran showed non-inferiority to warfarin in terms of safety but superiority in terms of efficacy. The Effective Anticoagulation with Factor Xa Next Generation in Atrial Fibrillation–Thrombolysis in Myocardial Infarction 48 (ENGAGE AF-TIMI 48) trial, a three-group, randomized, double-masked, double-dummy trial comparing two-dose regimens of edoxaban with warfarin. In 2740 patients with moderate kidney dysfunction, edoxaban showed no significant difference in efficacy but increased safety compared to warfarin [39]. Interestingly, Harrington et al. performed a meta-analysis including a total of 71,683 patients from individual patient data from the ROCKET AF, ARISTOTLE, RE-LY, and ENGAGE AF-TIMI 48 trials to assess the safety and efficacy of DOACs vs. warfarin across the continuous spectrum of CrCl [40]. The authors showed that using standard-dose DOACs is safer and more effective than warfarin for patients with a CrCl of at least 25 mL/min. Lower-dose DOACs do not significantly reduce the incidence of bleeding or intracranial hemorrhage compared to standard-dose DOACs, but they are associated with higher rates of death and stroke/systemic embolism. These findings advocate for the preference of standard-dose DOACs over warfarin in patients with impaired kidney function.

Less evidence is available on the DOAC and overall, on anticoagulant therapy in patients with atrial fibrillation and advanced kidney dysfunction. Indeed, among the randomized controlled trials, only ARISTOTLE included patients with CrCl < 30 mL/min [41]. It has been shown that in 269 patients with atrial fibrillation and advanced CKD, defined as a CrCl of 25–30 mL/min, apixaban has increased safety but not superior efficacy compared to warfarin. Despite the lack of evidence from randomized controlled trials, several observational studies explored the safety and efficacy of DOACs compared to VKAs in AF patients with advanced CKD [42,43,44]. Overall, these studies show similar efficacy in reducing stroke and systemic embolism and increased safety in reducing the bleeding risk of DOACs compared to VKAs. However, these studies have important limitations, including the low number of patients with stage V CKD. Thus, their findings must be interpreted with caution.

The Edoxaban Low-Dose for Elder Care Atrial Fibrillation Patients (ELDERCARE-AF) trial was a placebo-controlled trial comparing a once-daily 15 mg dose of edoxaban with a placebo in old Japanese patients with NVAF [45]. Among the entire cohort, 403 patients had a CrCl < 30mL/min. In these patients, the stroke or systemic embolism risk was decreased in those receiving edoxaban versus placebo, while no difference in major bleeding risk was detected.

The use of oral anticoagulants in CKD patients undergoing maintenance dialysis presents an even greater challenge than for those with advanced CKD not on dialysis. To date, dialysis patients have been excluded from all randomized controlled trials. However, since AF frequently occurs in these patients, anticoagulant therapy should be evaluated, taking into consideration that they have an increased risk of bleeding and mortality. Thus, clinicians must address the question of whether to perform anticoagulation therapy in AF patients on dialysis. All available evidence derives from observational studies. National and international clinical practice guidelines suggest that “it might be reasonable to prescribe warfarin or apixaban for dialysis patients with a CHA2DS2-VASc score greater than 2 in men and 3 in women”, providing a Class IIb recommendation due to the limited evidence available for this population [46]. Indeed, among all DOACs, apixaban is the least renally cleared, while warfarin is not renally cleared. Kuno et al. performed a meta-analysis to explore the safety and efficacy of DOACs and warfarin compared with no anticoagulants for AF patients on long-term dialysis [47]. The authors demonstrated that anticoagulants did not reduce the thromboembolism risk in AF patients on long-term dialysis. Additionally, warfarin, dabigatran, and rivaroxaban were linked to a significantly higher bleeding risk compared to apixaban and no anticoagulant. Four recent randomized controlled trials compared anticoagulants efficacy and safety in dialysis patients with AF. The Renal Hemodialysis Patients Allocated Apixaban Versus Warfarin in Atrial Fibrillation (RENAL-AF) was the first randomized controlled trial evaluating the safety or efficacy of apixaban compared to warfarin for stroke prevention in AF patients with end-stage kidney disease on hemodialysis [48]. However, the study was stopped prematurely due to enrollment issues. A sub-study on a small population of the RENAL-AF revealed that clinically relevant bleeding events were ≈10-fold more frequent than stroke or systemic embolism in this population. However, there is insufficient power to draw any conclusion on the safety of apixaban and warfarin in patients with AF and end-stage CKD on hemodialysis. Similarly, the AXADIA-AFNET 8 (Compare Apixaban and Vitamin K Antagonists in Patients With Atrial Fibrillation and End-Stage Kidney Disease) comparing apixaban and VKA in 97 patients with AF on hemodialysis failed to establish the non-inferiority or superiority of apixaban compared to warfarin [49].

In the Valkyrie trial, 132 AF patients on hemodialysis were randomized to VKA or rivaroxaban [50]. The authors showed that a reduced dose of rivaroxaban (10 mg daily) significantly reduced the rate of cardiovascular events and major bleeding complications compared with VKAs, suggesting a superior risk-benefit profile of DOACs versus VKAs in the hemodialysis population.

In conclusion, based on the current evidence from the literature, in patients with AF and moderate CKD, DOACs should be preferred over VKAs, as suggested by robust randomized controlled trial data. In patients with AF and advanced CKD (stages IV and V), less evidence is available (Table 1). In these patients, apixaban is often favored due to better pharmacokinetic properties, i.e., lower renal excretion. While apixaban is emerging as the most promising option, robust clinical trials are necessary to establish definitive guidelines. Until then, careful patient selection, close monitoring, and individualized therapy remain critical in managing this complex patient population.

Emerging evidence supports low-dose DOACs as a viable alternative in selected patients with advanced CKD. Low-dose DOACs, particularly apixaban, are emerging as a safe and effective option for both therapeutic anticoagulation and long-term prophylaxis in patients with advanced CKD, including those on dialysis. While data remains limited compared to patients with normal renal function, real-world studies and pharmacokinetic modeling support their cautious use. Individualized assessment of bleeding vs. thrombotic risk remains essential, and ongoing prospective studies are warranted to refine dosing strategies in this high-risk group.

### 3.3. DOACs in Nephrotic Syndrome

Nephrotic syndrome (NS) is a common kidney disease affecting both children and adults. It is characterized by significant proteinuria (>3.5 g/day), hypoalbuminemia, and edema. In children, minimal change disease (MCD) is the most common cause, while in adults, membranous nephropathy (MN), focal segmental glomerulosclerosis (FSGS), and MCD are prevalent.

The glomerular filtration barrier dysfunction in NS leads to the loss of plasma proteins, including albumin and anticoagulants. In response, the liver increases the production of procoagulation factors, contributing to a hypercoagulable state. Platelet aggregation and reduced fibrinolysis further increase the risk of thromboembolic events, which can be as high as 25%, particularly in MN within the first six months.

Since thromboembolic complications significantly increase morbidity and mortality, prophylactic anticoagulation is recommended, especially in patients with severe hypoalbuminemia (<2.4 g/dL in MN and <2 g/dL in other forms). Current KDIGO guidelines favor heparin and VKAs due to their established use in NS and other conditions. However, emerging reports, including case series and small studies, suggest potential benefits of DOACs in NS, though data remains limited [31]. A Danish study on 21 NS patients using DOACs found no thromboembolic events and only minor bleeding episodes [51]. Van Meerhaeghe et al. studied 24 patients on apixaban for primary prophylaxis, with a mean treatment duration of 129 days [52]. Only one patient developed pulmonary embolism, and no major or minor bleeding events were reported. Similarly, in the case series of 11 patients with NS and severe hypoalbuminemia described by Nissar et al., no bleeding complications were observed, though one patient experienced thrombus progression on apixaban and required a switch to VKA [53]. Sexton et al. presented two cases where apixaban was used prophylactically against thromboembolism in NS patients. The authors also reviewed the existing literature and concluded that, despite limited data, initial experiences with DOACs in NS appear promising [54]. Tijani et al., in a retrospective study, found that the use of DOACs for VTE prophylaxis was associated with a lower rate of major bleeding compared to warfarin, though the difference was not statistically significant but clinically meaningful [55]. Similarly, El-Bardissy et al. performed a retrospective cohort study comparing the safety and efficacy of DOACs versus warfarin for VTE prophylaxis in patients with NS. They reported that DOACs may be as effective as warfarin for VTE prophylaxis in NS patients while exhibiting a lower risk of non-major bleeding [56].

Concerns persist regarding DOACs in NS due to their high protein binding (90%), which raises questions about overexposure (increased free drug levels leading to bleeding) or underexposure (urinary drug loss reducing efficacy). A pharmacokinetic study is currently underway (NCT04278729) to clarify DOAC behavior in NS patients [57]. Until more evidence emerges, LMWH and warfarin remain the preferred options for VTE prophylaxis in NS, while DOACs may be considered in select cases (Table 1). Further large-scale studies are required to determine the optimal anticoagulation regimen in NS patients. The KDIGO 2024 guidelines emphasize the need for better risk stratification to assess disease-specific thrombotic risk, multicenter observational registries to track VTE incidence in NS patients, pharmacokinetic studies on DOACs to determine their safety and efficacy in NS, and RCTs comparing DOACs, LMWH, and warfarin [31]. Additionally, the efficacy of low-dose DOACs in NS is currently under investigation. Early data suggest they may be a reasonable option for prophylaxis and treatment of thrombosis in carefully selected patients, particularly those with preserved renal function and low bleeding risk. Thus, larger, controlled studies are needed to establish safety, efficacy, and optimal dosing in this unique population.

### 3.4. DOACs in Cancer

**Figure 3:**
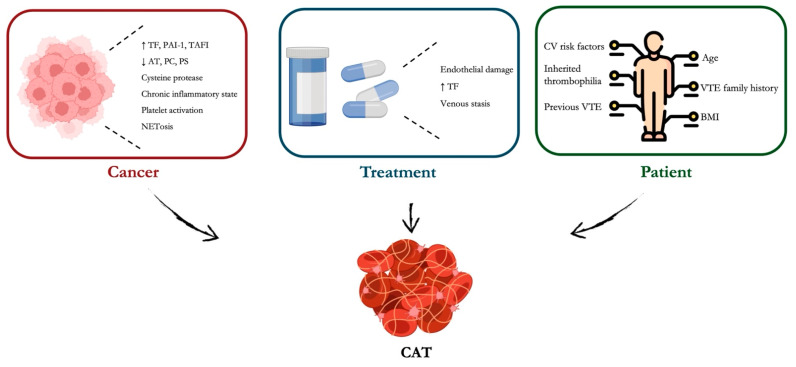
Pathological mechanisms underpinning cancer-associated thrombophilia (CAT).

Cancer-associated thrombosis (CAT) is a frequent complication in cancer patients, primarily involving PE and DVT in both the upper and lower extremities [58]. It has been reported that cancer patients have a 12-fold higher risk of VTE compared to the general population, and this risk increases up to 23-fold in those receiving systemic anticancer therapies [59]. Thromboembolism is the second leading cause of death in cancer patients and contributes significantly to morbidity, delayed treatments, healthcare costs, and reduced quality of life [60,61]. The mechanisms underpinning CAT are various and complex. They can be grouped into three categories: (i) patient-related, including age, previous and family history of VTE, cardiovascular risk factors, low and high BMI, and inherited thrombophilia; (ii) tumor-related, including the type, i.e., gastrointestinal (GI), brain, lung and genitourinary are associated with an increased CAT risk, the histological tumor grade, with a high grade being associated with an increased risk, and the time since diagnosis. Additionally, cancer-induced inflammation can lead to platelet activation and clot formation; and (iii) treatment-related. Chemotherapy and major cancer surgeries can induce endothelial damage [58] (Figure 3). Drugs like cisplatin, thalidomide, and bevacizumab are known to increase thrombotic risk through endothelial injury and enhanced TF expression [62]. Also, central venous catheters frequently used in cancer patients may disrupt venous endothelium and serve as a nidus for clot formation [63]. Finally, cancer-related fatigue, hospitalizations, and surgical interventions increase the risk of venous stasis, one of the three components of Virchow’s triad (stasis, endothelial injury, and hypercoagulability [64].

Cancer cells, especially pancreatic, brain, and lung, frequently overexpress tissue factor (TF), which interacts with factor VIIa, leading to the activation of factor X and downstream thrombin generation [65]. Additionally, malignant cells uniquely produce cysteine protease (CP), which directly activates factor X independent of factor VIIa or TF [66]. Though less studied, CP contributes to thrombin generation and fibrin formation. Cancer promotes a chronic inflammatory state characterized by elevated IL-1, IL-6, and TNF-α, which upregulate TF expression and downregulate anticoagulant pathways [67]. Moreover, endothelial activation or injury leads to increased adhesion molecule expression, such as P-selectin, favoring leukocyte and platelet binding and enhancing thrombus formation. Tumor cells interact and activate platelets via direct binding or release of platelet-activating factors, such as ADP, thromboxane A2 [68]. Another cancer-related mechanism underpinning CAT is NETosis. It is a unique form of cell death characterized by the release of decondensed chromatin and granular contents to the extracellular space. In cancer, NETosis is often dysregulated, contributing to immunothrombosis through activation of platelets, release of histones and DNA, which can directly activate coagulation, and activated platelets, which facilitate tumor growth and metastasis and provide a procoagulant surface for clotting reactions [69,70].

Many tumors upregulate plasminogen activator inhibitor-1 (PAI-1), which inhibits tissue plasminogen activator (tPA), reducing fibrin degradation [71]. This imbalance between coagulation and fibrinolysis further tips the scale toward a prothrombotic state. Cancer may also disrupt natural anticoagulant pathways by downregulating antithrombin, protein C, and protein S, and increasing the expression of inhibitors like PAI-1 and thrombin-activatable fibrinolysis inhibitor (TAFI).

Thus, thrombosis in cancer is a result of a complex interaction between tumor biology, host immune and coagulation systems, and external factors such as therapy and immobilization.

The elevated incidence of VTE in cancer patients and its substantial effect on morbidity and mortality have led to a growing interest in using anticoagulants for primary VTE prevention. However, the management of CAT is challenging due to increased bleeding risks and drug interactions. Traditionally, LMWH has been the standard of care, but the use of DOACs is becoming more prevalent due to their ease of administration and favorable safety profile.

Several RCTs investigating treatments for cancer-associated VTE have shown that parenteral anticoagulation, such as LMWH, reduces the risk of recurrent VTE without increasing the risk of major bleeding compared to VKA therapy [72]. Recent studies indicate that DOACs are equally effective as parenteral anticoagulation in preventing recurrent VTE.

In 2018, the Hokusai VTE Cancer trial compared edoxaban with subcutaneous dalteparin to treat patients with CAT [73]. The authors found that Edoxaban was non-inferior to dalteparin for recurrent VTE prevention. However, the rate of major bleeding was higher with edoxaban than with dalteparin. In 2019, the Selected Cancer Patients at Risk of Recurrence of Venous Thromboembolism (SELECT-D) Trial compared rivaroxaban vs. dalteparin for 6-month VTE treatment in cancer patients [74]. There is a higher risk of clinically relevant non-major bleeding (CRNMB) with edoxaban, especially in GI cancers. Rivaroxaban significantly reduced recurrent VTE (4% vs. 11% in dalteparin) while increasing GI bleeding risk, particularly in upper GI cancers. In 2021, the Apixaban for the Treatment of Venous Thromboembolism in Patients With Cancer (ADAM VTE) Trial evaluated apixaban vs. dalteparin in cancer patients with VTE [75]. Apixaban was associated with lower major bleeding rates and fewer recurrent VTE events compared to dalteparin. In 2020, the CARAVAGGIO trial compared apixaban vs. dalteparin in cancer patients with symptomatic or incidental VTE [76]. Apixaban was non-inferior to dalteparin for VTE recurrence and showed no significant increase in major bleeding, even in GI cancers.

Fjisaki et al. performed a meta-analysis including 17 RCTs involving 6623 patients with active cancer [77]. The authors found that all DOACs demonstrated similar efficacy in preventing recurrent VTE. However, their safety profiles varied: apixaban was linked to a lower risk of major bleeding compared to edoxaban, while edoxaban had a reduced risk of clinically relevant nonmajor bleeding (CRNMB) compared to rivaroxaban. Compared to parenteral anticoagulation, apixaban was associated with a lower risk of recurrent VTE without increasing bleeding risks, whereas edoxaban and rivaroxaban had higher bleeding risks. The study underscores the complex interplay of cancer-related, patient-specific, and treatment-related factors that influence thrombotic and bleeding risks in cancer patients, making anticoagulation management particularly challenging.

Guidelines recommend the use of DOACs in cancer patients. The ISTH Guidelines recommend DOACs (apixaban, edoxaban, rivaroxaban) over LMWH for CAT treatment except in patients with high GI bleeding risk [78]. The American Society of Clinical Oncology (ASCO) Guidelines prefer DOACs over LMWH in most cancer patients unless contraindicated due to bleeding risk [79]. The National Comprehensive Cancer Network (NCCN) Guidelines recommend DOACs as an alternative to LMWH for CAT management, except in GI and genitourinary cancers where bleeding risk is higher [80]. Finally, the latest 2022 European Society of Cardiology guidelines for cardio-oncology endorse the use of apixaban, edoxaban, rivaroxaban, and parenteral therapy for treating cancer-associated thrombosis [81,82].

Overall, DOACs have several advantages for CAT treatment, including the oral administration, which eliminates the need for injections and thus improves patient compliance; the fixed dosing, which eliminates the need for routine monitoring; and the lower risk of heparin-induced thrombocytopenia (hit) in contrast to LMWH. While standard doses are recommended for acute treatment, low-dose DOACs are increasingly studied for extended prophylaxis after the initial treatment phase. Low-dose DOACs, particularly apixaban and rivaroxaban, show promise for long-term prophylaxis in cancer patients at continued risk of thrombosis, especially after completing initial treatment. They may reduce VTE recurrence with an acceptable safety profile, but patient selection is key. Ongoing trials will help define optimal dosing strategies for extended use.

Despite their advantages, DOACs must be used cautiously in cancer due to (i) increased bleeding risk, especially in GI malignancies (e.g., stomach, pancreatic cancers); (ii) drug interactions. Indeed, many chemotherapy agents (e.g., tyrosine kinase inhibitors, proteasome inhibitors) interact with DOACs via CYP3A4 and p-glycoprotein metabolism, affecting their efficacy and safety; (iii) renal and hepatic dysfunction. DOACs are partially renally excreted (e.g., edoxaban, rivaroxaban), requiring dose adjustments in patients with renal impairment. Additionally, liver metastases or chemotherapy-induced hepatotoxicity can affect DOAC metabolism; (iv) their use should be limited in patients with brain tumors because they are at increased risk of intracranial hemorrhage, making DOACs less favorable (Table 1).

### 3.5. DOACs in Obesity

Obesity, defined as a body mass index (BMI) of 30 kg/m^2^ or higher, is a complex and growing global public health issue that has reached epidemic levels. Projections indicate that the prevalence of obesity will increase significantly from 14% to 24% by 2035, affecting nearly 2 billion people worldwide [83]. Notably, obesity is associated with a 6.2-fold increased risk for VTE and is a risk factor for developing AF [84]. The Framingham Heart Study has revealed that each unit increase in BMI is associated with a 4–5% higher risk of developing AF [85]. Additionally, a meta-analysis found that a 5-unit increase in BMI corresponds to a 10–29% greater risk of incident, postoperative, and post-ablative AF [86]. However, anticoagulant therapy in obese patients is challenging because obesity may influence the pharmacokinetics of drugs by altering their volume of distribution (Vd), peak concentration (Cmax), drug exposure, and drug clearance [87,88]. Additionally, since DOACs are administered at fixed doses without routine monitoring, concerns arise about whether standard dosing is sufficient in severe obesity.

Comprehensive data on the clinical pharmacokinetics, pharmacodynamics, efficacy, and safety of DOACs in individuals with obesity are limited, largely due to the significant underrepresentation of this patient population in key clinical trials. Historically, the use of DOACs in obese patients has been discouraged. In 2016, the ISTH Recommendation cautioned against DOAC use in patients with a BMI > 40 kg/m^2^ or weight > 120 kg due to limited data. Subsequently, ISTH now endorses these agents at standard doses for VTE prevention and management in patients with a BMI > 40 kg/m^2^ or weight > 120 kg [89]. This recommendation is further supported by studies showing that anti-Xa levels in obese patients are not significantly different from those in individuals with a normal BMI [90]. However, ISTH still advises against the use of dabigatran, edoxaban, and betrixaban due to insufficient supporting data. A recent retrospective study from the VENUS network also indicated that DOACs are associated with lower rates of major bleeding compared to warfarin in obese patients treated for VTE [91]. Similarly, there is evidence supporting the use of DOACs for AF in obese individuals. A recent review concluded that DOACs demonstrated better efficacy and safety than warfarin across all BMI categories, including those with morbid obesity, aligning with previous studies [92]. Nevertheless, the European Society of Cardiology (ESC) warns that DOAC safety and efficacy data become less reliable in patients with a BMI exceeding 40 kg/m^2^. For those with a BMI over 50 kg/m^2^, therapeutic drug monitoring or switching to a VKA may be a more appropriate approach [93].

Among DOACs, apixaban and rivaroxaban have demonstrated stable pharmacokinetic profiles in obese individuals, whereas dabigatran has shown greater variability in drug levels [94,95].

Current evidence does not support routine dose adjustments for most DOACs in obesity. However, TDM can be considered in extreme obesity [89]. Apixaban and rivaroxaban are preferred due to their robust clinical data on obesity. Anti-Xa levels (for rivaroxaban and apixaban) and thrombin time (for dabigatran) may be used in select cases, though routine monitoring is not standard [96].

Many real-world studies have shown DOACs to be effective and safe in obese populations, but large-scale randomized trials are still needed for definitive conclusions (Table 1).

While DOACs remain a viable anticoagulation option for obese patients, careful consideration should be given to drug selection, pharmacokinetics, and potential monitoring in extreme obesity. Current data suggest that rivaroxaban and apixaban are the most reliable DOAC choices for this population, whereas dabigatran and edoxaban should be used cautiously. Ongoing research will further clarify the optimal anticoagulation strategy in obesity, ensuring both efficacy and safety in this high-risk group. Evidence supports cautious use of low-dose DOACs, e.g., apixaban 2.5 mg BI, for extended VTE prophylaxis in selected obese patients, particularly those with stable weight and no other high-risk features. However, due to variability in absorption and clearance, standard doses are preferred for initial treatment, and monitoring may be considered in extreme obesity.

### 3.6. DOACs in Antiphospholipid Antibody Syndrome

Antiphospholipid syndrome (APS) is an autoimmune disorder characterized by recurrent arterial and/or venous thrombosis and pregnancy-related complications [97]. Traditionally, VKAs have been the mainstay of anticoagulation therapy in APS patients. The emergence of DOACs has prompted interest in their potential use for APS. However, several challenges and considerations have been recognized. Evidence indicates that DOACs may be associated with an increased risk of arterial thrombotic events in APS patients. A meta-analysis of RCTs found that patients with thrombotic APS randomized to DOACs had increased odds of arterial thrombosis compared to those receiving VKAs, particularly strokes [98]. Additionally, the TRAPS (rivaroxaban in Thrombotic Antiphospholipid Syndrome) study reported that rivaroxaban was less effective than warfarin in preventing thrombotic events in high-risk APS patients, specifically those with triple-positive antibody profiles [99]. This led to the premature termination of the trial due to an excess of thrombotic events in the rivaroxaban group. In a recent randomized controlled trial, Ordi-Ros and colleagues were unable to establish that rivaroxaban 20 mg daily was non-inferior to VKAs in a cohort of 190 adults with VTE or arterial thrombotic APS [100]. Several observational studies and case series have reported mixed results regarding the efficacy and safety of DOACs in APS patients, leading to uncertainty about their role in this population [101].

There remains a pressing need for further research to assess the efficacy and safety of DOACs in APS patients. Notably, randomized trials evaluating DOACs in VTE did not screen for antiphospholipid antibodies and systematically excluded patients with known APS. Given the heterogeneity of APS, the risk of recurrent thrombosis may vary based on factors such as antibody titers, lupus anticoagulant positivity, triple positivity, and whether the initial thrombotic event was arterial or venous. While findings from the TRAPS trial and the Ordi-Ros study raise concerns about the effectiveness of rivaroxaban compared to VKAs in APS, it remains unclear whether these results apply to all APS subgroups or extend to other DOACs.

Over the years, international thrombosis and cardiology societies have provided discordant recommendations on the use of DOACs in patients with APS.

The 2019 ESC guidelines advise against the use of DOACs in patients with APS, without distinguishing between different DOACs, types of APS (venous or arterial), or levels of antibody positivity (single, double, or triple positive). However, this recommendation is primarily based on the TRAPS trial, which included triple-positive APS patients with both prior venous and arterial thrombotic events, comparing rivaroxaban with conventional treatment. Similarly, the 2020 International Society on Thrombosis and Haemostasis (ISTH) guidelines do not recognize DOACs as a valid treatment option for any APS patient, except for low-risk, stable patients already receiving DOAC therapy, who may continue treatment after shared informed decision-making [102]. In contrast, the 2019 European League Against Rheumatism (EULAR) guidelines provide a more nuanced recommendation, considering APS patient subtypes based on their clinical phenotype [97]. While DOACs, particularly rivaroxaban, are contraindicated in triple-positive APS patients and those with arterial thrombosis, their use may be considered in venous APS patients without triple positivity. Additionally, EULAR acknowledges that some patients may have poor quality warfarin therapy (low time in therapeutic range, TiTR) or intolerance to VKAs, in which case DOACs may be an alternative. The 2020 British Society of Haematology (BSH) guidelines take a similar approach, recommending against DOAC use in arterial APS patients [103]. However, for venous APS patients, including both triple-positive and non-triple-positive cases already on DOACs, treatment may be continued if the patient refuses to switch to VKAs.

The 2020 American Society of Hematology (ASH) guidelines state that APS patients are not ideal candidates for DOAC therapy. In cases of recurrent thrombosis while on VKA treatment, LMWH is preferred over DOACs. However, ASH acknowledges that this recommendation is based on very low-certainty evidence [104]. Additionally, evidence for using low-dose DOACs (e.g., rivaroxaban 10 mg) is limited and generally not recommended, especially in high-risk patients (triple positive). Observational data suggest that low-dose DOACs may be considered in low-risk APS patients (who cannot tolerate warfarin). However, no randomized trials have specifically supported low-dose DOACs for long-term prophylaxis in APS.

Overall, while most guidelines discourage DOAC use in high-risk APS patients (triple-positive or arterial APS), there is some allowance for venous APS patients without triple positivity, particularly in cases of VKA intolerance or poor therapeutic control. The lack of consensus across guidelines stems from the scarcity of randomized trials and the absence of rigorous patient stratification, highlighting the need for further research in this area [101].

In conclusion, current evidence and guideline recommendations generally advise against the use of DOACs in APS patients, especially those with high-risk profiles, due to concerns about increased arterial thrombotic events (Table 1). VKAs remain the preferred anticoagulant therapy for most APS patients. In situations where VKAs are not suitable, such as intolerance or inability to maintain therapeutic INR, the potential use of DOACs should involve careful consideration of individual patient risk factors and preferences, with a thorough discussion of the associated risks and benefits.

### 3.7. DOACs in Embolic Stroke of Undetermined Source

Embolic Stroke of Undetermined Source (ESUS) is a subtype of ischemic stroke where no clear embolic origin, such as AF, significant carotid stenosis, or other major cardioembolic sources, is identified after a standard diagnostic workup [105]. ESUS accounts for approximately 20–25% of ischemic strokes and is believed to be caused by cryptogenic embolism, possibly from occult AF, nonstenotic atherosclerotic plaques, or other undiagnosed cardioembolic sources. Consequently, there is a clear need for effective therapeutic strategies for secondary prevention in these patients [106].

Given the embolic nature of ESUS, anticoagulation has been proposed as a superior preventive strategy compared to antiplatelet therapy, with DOACs emerging as a potential alternative to aspirin in secondary stroke prevention. However, large clinical trials evaluating DOACs in ESUS have yielded mixed results, leading to an ongoing debate regarding their role in treatment. The three major RCTs are the NAVIGATE ESUS, RE-SPECT ESUS, and the ATTICUS. The NAVIGATE ESUS compared rivaroxaban (15 mg daily) with aspirin (100 mg daily) in 7213 patients with recent ESUS [105]. No significant reduction in recurrent stroke was observed with rivaroxaban vs. aspirin (4.7% vs. 4.8% annual event rate), while an increased risk of major bleeding in the rivaroxaban group (1.8% vs. 0.7% with aspirin) was found. Thus, the authors concluded that rivaroxaban was not superior to aspirin and led to a higher bleeding risk.

The RE-SPECT evaluated dabigatran (150 mg twice daily) vs. aspirin (100 mg daily) in 5390 ESUS patients [107]. The authors found that Dabigatran did not significantly reduce stroke recurrence (4.1% vs. 4.8% per year), but subgroup analyses suggested a possible benefit in older patients (>75 years) and those with atrial cardiopathy. Major bleeding rates were similar between dabigatran and aspirin. In conclusion, DOACs showed no overall benefit but potential advantages in select subgroups. Finally, the ATTICUS investigated apixaban vs. aspirin in ESUS patients with atrial cardiopathy [108]. Preliminary data suggest no significant stroke reduction compared to aspirin.

Notably, the ESUS population is diverse, involving multiple underlying mechanisms such as atherosclerosis, cardioembolism, and coagulation abnormalities, indicating that a single treatment approach may not be effective for all patients [109].

Currently, aspirin remains the standard of care for secondary stroke prevention in ESUS, as DOACs have not demonstrated superiority in the general ESUS population. DOACs may be considered in specific ESUS subgroups, such as patients with atrial cardiopathy, patients with covert AF detected on extended cardiac monitoring, and patients with high-risk aortic plaques or left ventricular dysfunction.

The failure of clinical trials evaluating the efficacy of DOACs in patients with ESUS necessitates a broader consideration of its classification. Indeed, the heterogeneity within the ESUS population may influence the effectiveness of anticoagulant therapy. ESUS encompasses a variety of pathophysiological mechanisms, including occult cardioembolic sources, patent foramen ovale, non-stenotic atherosclerotic plaques, and other prothrombotic conditions [110]. This heterogeneity implies that not all ESUS subtypes benefit equally from anticoagulation therapy [109]. For instance, in patients with PFO, anticoagulation has been shown to significantly reduce the risk of ischemic stroke compared to antiplatelet therapy. Conversely, anticoagulation has not demonstrated a significant advantage over antiplatelet agents in other subgroups.

Therefore, a more personalized approach to the management of ESUS patients is warranted, with a focus on identifying specific pathophysiological mechanisms through comprehensive diagnostic evaluation (Table 1). This strategy would facilitate the selection of the most appropriate therapy for each patient, ultimately improving clinical outcomes and reducing the risk of recurrent stroke.

### 3.8. DOACs in Superficial Venous Thrombosis

Superficial venous thrombosis (SVT), also known as superficial thrombophlebitis, is characterized by clot formation in the superficial veins, typically affecting the lower extremities [111]. While traditionally considered a benign and self-limiting disorder, growing evidence suggests that SVT is associated with a significant risk of thromboembolic complications, including DVT and PE. Thus, anticoagulant therapy has been explored as a treatment option, with DOACs emerging as potential alternatives to traditional therapies.

SVT can result from venous stasis, endothelial injury, or hypercoagulability [112]. Conditions such as varicose veins, thrombophilia, malignancy, recent surgery, pregnancy, or immobilization can increase the risk of SVT. Studies indicate that up to 25% of SVT cases may be associated with concurrent DVT or PE, underscoring the need for appropriate risk stratification and treatment [113].

The management of SVT depends on its extent, location, and associated risk factors. Historically, treatment options have included supportive therapy, such as compression stockings and mobilization, for mild, localized SVT; anticoagulation with LMWH or fondaparinux for high-risk cases, particularly when the thrombus is ≥5 cm in length or located near the saphenofemoral junction; and surgical interventions with vein ligation or stripping in selected cases of recurrent or extensive thrombosis. Limited evidence is available on the effectiveness of DOACs in treating SVT.

In 2017, the SURPRISE Trial compared rivaroxaban (10 mg daily) with fondaparinux (2.5 mg daily) in 472 patients with SVT ≥ 5 cm. The authors found that rivaroxaban was non-inferior to fondaparinux in preventing thromboembolic events. Interestingly, neither group experienced major bleeding, suggesting that rivaroxaban, being less expensive, could be a reliable alternative to fondaparinux in high-risk SVT patients [114]. In 2020, Kearon et al. evaluated rivaroxaban vs. placebo in patients with leg SVT of at least 5 cm [115]. The authors did not find differences in efficacy or safety between rivaroxaban and placebo in patients with symptomatic SVT. Current guidelines do not recommend the use of DOACs in SVT patients due to the limited evidence available. American College of Chest Physicians (ACCP) guidelines recommend fondaparinux or LMWH for 45 days in high-risk SVT and suggest that DOACs may be a reasonable alternative in select cases, although more evidence is needed [116]. The ESC Guidelines recognize the role of DOACs in VTE prevention but do not yet formally recommend them for SVT due to limited data [117]. Finally, the ISTH suggests that DOACs could be an alternative to fondaparinux, especially for patients preferring an oral regimen [118].

While DOACs are not yet the first-line treatment for SVT, they offer a promising oral alternative to injectable anticoagulants [119] (Table 1). Potential scenarios where DOACs may be considered include patients with extensive SVT (≥5 cm) or proximity to deep veins who require anticoagulation but prefer oral therapy; patients with contraindications or intolerance to injections (e.g., LMWH, fondaparinux); patients with SVT and additional thrombotic risk factors (history of DVT, malignancy, thrombophilia); and long-term secondary prevention in patients with recurrent SVT [120].

DOACs such as rivaroxaban and apixaban show promise as an alternative to fondaparinux and LMWH in the treatment of high-risk SVT, offering oral convenience and comparable efficacy. However, larger trials are needed to establish their role in standard guidelines. For now, fondaparinux remains the preferred anticoagulant, but DOACs may be a viable option for selected patients requiring anticoagulation. Currently, the decision to use DOACs in SVT should be individualized, considering patient preference, bleeding risk, and the extent of thrombosis. Finally, current evidence supports low-dose DOACs for short-term therapeutic anticoagulation in SVT. Data on long-term prophylaxis are limited, and extended use should be individualized.

### 3.9. DOACs in Upper Extremity Deep Vein Thrombosis

Upper extremity deep vein thrombosis (UEDVT) refers to thrombosis in the deep veins of the upper limbs, most commonly affecting the axillary and subclavian veins [121]. While UEDVT accounts for approximately 5–10% of all DVT cases, its incidence is increasing, largely due to the more frequent use of intravenous catheters and other medical devices. UEDVT can be classified into primary and secondary forms. Primary UEDVT, also known as Paget–Schroetter syndrome, is caused by repetitive upper limb movements in a patient with thoracic outlet compression. Secondary UEDVT is more prevalent and typically results from indwelling devices such as central venous catheters, pacemakers, or defibrillator leads. The presence of a central venous catheter increases the risk of developing UEDVT by up to 14-fold [122].

Patients with UEDVT may present with symptoms including pain, swelling, redness, and a sensation of heaviness in the affected limb. Prompt diagnosis is crucial to prevent complications such as PE and post-thrombotic syndrome (PTS). Current guidelines recommend anticoagulation for UEDVT for at least three months. The treatment choice depends on the patient’s risk factors and clinical presentation. Specifically, for catheter-related UEDVT, anticoagulation is recommended as long as the catheter remains in place, and for at least three months after removal; for effort thrombosis or idiopathic UEDVT, three to six months of anticoagulation is typically recommended; and for malignancy-associated UEDVT, extended anticoagulation with DOACs or low-molecular-weight heparin (LMWH) is advised.

While extensive research supports the use of DOACs for lower extremity DVT, their efficacy and safety in UEDVT remain unclear, as such events were excluded from clinical trials leading to DOAC approval. Indeed, data on DOACs in UEDVT derive from anecdotal evidence, analysis of registries, and small single-center studies [123,124]. Garcia et al. performed a retrospective analysis showing that apixaban and rivaroxaban were associated with high thrombus resolution rates and a reduced risk of recurrent UEDVT [125]. Young et al. found that patients treated with apixaban for UEDVT had comparable clinical outcomes to those treated with VKAs, with improved adherence and fewer complications [126]. Konstantinides et al. conducted a multicenter trial comparing rivaroxaban with warfarin in patients with UEDVT and found that the recurrence rate of VTE was similar in both groups, with a slight reduction in bleeding events in the DOAC group [127]. The meta-analysis conducted by Houghton et al. revealed that DOACs were associated with a lower risk of major bleeding compared to warfarin [124]. Carrier et al. found that the use of rivaroxaban and apixaban was associated with a reduced incidence of intracranial and gastrointestinal bleeding, making them a safer alternative for long-term anticoagulation in UEDVT patients [128]. No evidence is available on the efficacy and safety of low-dose DOACs for initial treatment or long-term prophylaxis in UEDVT.

While current evidence indicates that DOACs are an optimal option with comparable efficacy and improved safety profiles in the treatment of UEDVT compared to traditional anticoagulation therapies, further large-scale randomized controlled trials are warranted to optimize treatment protocols and evaluate long-term outcomes (Table 1). Clinicians should consider individual patient factors, including the presence of indwelling devices and potential drug interactions, when selecting an anticoagulant regimen for UEDVT management. Indeed, potential drug interactions with P-gp and CYP3A4 inhibitors have been reported [129].

### 3.10. DOACs in Inferior Vena Cava Thrombosis

Inferior vena cava thrombosis (IVCT) is a rare but serious clinical condition characterized by thrombus formation in the superior or inferior vena cava and is associated with high morbidity [130]. The precise incidence of IVC thrombosis is challenging to determine due to its heterogeneous clinical presentations, including leg heaviness, pain, swelling, and cramping. Among patients diagnosed with DVT, the reported occurrence of associated IVCT ranges from 4% to 15%. However, the actual prevalence may be underestimated due to variations in symptomatology and presentation. Case reports suggest that IVCT may be underdiagnosed. Typically, pulmonary emboli are believed to originate from DVT in the lower extremities. However, some cases have demonstrated that an IVCT was the source of the detected PE. Therefore, IVC thrombosis should be included in the differential diagnosis for patients at risk of thromboembolic events. Once an IVC thrombus is identified, it is crucial to determine the underlying prothrombotic condition, as multiple potential causes exist [131,132]. Historically, anticoagulation with VKAs and LMWH has been the cornerstone of treatment. However, DOACs have emerged as an effective and potentially safer alternative. Rodriguez et al. evaluated the use of apixaban and rivaroxaban in malignancy-associated IVCT and found that these agents had a similar efficacy profile to LMWH but with improved patient adherence [133]. Prandoni et al. conducted a prospective cohort study comparing DOACs with VKAs in IVCT patients, showing similar rates of thrombus resolution and recurrent VTE between the two groups, and, thus, supporting the non-inferiority of DOACs [134]. Additionally, Wiggins et al. performed a retrospective analysis of patients with IVC thrombosis treated with DOACs and observed a significant reduction in recurrent thrombotic events compared to warfarin, with comparable efficacy [135]. Carrier et al. reported a lower incidence of major bleeding complications in patients with IVC thrombosis treated with DOACs compared to VKAs [117]. Kearon et al. emphasized that the predictable pharmacokinetics of DOACs eliminate the need for frequent monitoring, reducing the risk of supratherapeutic anticoagulation and bleeding events [136]. In conclusion, DOACs have demonstrated comparable efficacy and a favorable safety profile in the treatment of IVC thrombosis, making them a viable alternative to traditional anticoagulation therapies. While further randomized controlled trials are needed to establish definitive protocols, current evidence supports the use of DOACs in these patients (Table 1). Finally, there is no direct evidence supporting low-dose DOACs for either initial treatment or long-term prophylaxis in IVC thrombosis. Management typically follows proximal DVT protocols, with standard-dose DOACs preferred and long-term decisions individualized.

### 3.11. DOACs in Pelvic Vein Thrombosis in Women

Pelvic vein thrombosis (PeVT) is a rare but potentially serious form of VTE involving thrombus formation in the pelvic veins, including the iliac veins, ovarian veins, and other tributaries. It is a rare disorder with a prevalence of 0.05 to 3% [137]. Several factors contribute to the development of PeVT, including pregnancy and the postpartum period, cancers, surgical procedures, inflammatory conditions, such as pelvic inflammatory disease or inflammatory bowel disease, and inherited or acquired hypercoagulable states, such as FV Leiden, antiphospholipid syndrome, and protein C or S deficiencies. The symptoms of pelvic vein thrombosis can vary widely, but common presentations include lower abdominal or pelvic pain, fever (especially in postpartum cases), swelling and tenderness in the lower extremities, tachycardia and respiratory symptoms in cases of pulmonary embolism, and unilateral leg swelling if associated with iliac vein involvement. Prompt diagnosis is essential to prevent complications and start the treatment focused on preventing clot propagation, recurrence, and embolic complications. Due to its uncommon occurrence, specific studies focusing solely on PeVT are limited. However, insights can be drawn from research on similar conditions, such as splanchnic vein thrombosis (SVT) and gynecological cancer-associated VTE, to evaluate the safety and efficacy of DOACs in treating PeVT. A comprehensive review by Riva et al. explored the use of DOACs in patients with unusual site VTEs, including SVT and cerebral vein thrombosis (CVT) [118]. The study found that DOACs demonstrated comparable effectiveness to traditional VKAs and were associated with a trend toward better safety profiles. However, the authors emphasized the need for caution in special patient populations, such as those with liver cirrhosis or malignancy, due to the limited and low-quality evidence available. Shimizu et al. conducted a retrospective study focusing on patients with gynecological cancers who developed VTE, including cases involving pelvic veins [138]. The study compared DOACs to VKAs and found that DOACs were non-inferior in terms of efficacy and safety. Specifically, recurrent VTE occurred in 5.7% of patients in the VKA group compared to none in the DOAC group, while clinically relevant bleeding was observed in 1.9% of the DOAC group. These findings suggest that DOACs can be effectively and safely used in VTE patients with gynecological cancers. While direct studies on DOAC use specifically for PeVT are scarce, the extrapolated data from related conditions suggest that DOACs may offer a safe and effective alternative to traditional anticoagulants (Table 1). However, clinicians should use caution, especially in patients with underlying conditions such as liver disease or active malignancy. Individual patient factors, potential drug interactions, and the specific characteristics of the thrombosis should guide the choice of anticoagulant therapy. Based on data extrapolated from extended VTE trials, low-dose DOACs are not suitable for initial treatment of pelvic vein thrombosis but may be considered for long-term prophylaxis in women at persistent risk.

### 3.12. DOACs in Cerebral Venous Thrombosis

Cerebral venous thrombosis (CVT) is an uncommon cerebrovascular disorder characterized by the formation of thrombi within the dural sinuses and cerebral veins. It accounts for a small percentage of all strokes but predominantly affects younger individuals, especially women. The clinical presentation is highly variable, ranging from isolated headaches to severe neurological deficits. Prompt diagnosis and effective anticoagulation are crucial to prevent complications and improve outcomes. The RE-SPECT CVT trial was a pivotal RCT comparing dabigatran with warfarin in 120 patients with CVT [138]. The study demonstrated that dabigatran was non-inferior to warfarin in terms of efficacy, with similar rates of recurrent VTE and major bleeding events. However, the trial’s sample size limited its power to detect rare adverse events. A systematic review and meta-analysis encompassing both RCTs and observational studies suggested that DOACs have comparable efficacy and safety profiles to VKAs in the treatment of CVT [139]. The analysis reported similar risks of recurrent VTE, major hemorrhage, and death between the two anticoagulant classes. The current body of evidence indicates that DOACs are a viable alternative to VKAs for the treatment of CVT. Their use is associated with comparable efficacy in preventing recurrent thrombotic events and a potentially improved safety profile concerning major bleeding complications. The convenience of fixed dosing and the absence of routine monitoring make DOACs attractive in clinical practice. However, limitations exist, including the relatively small sample sizes of RCTs and the retrospective nature of observational studies, which may introduce bias. Finally, a recent open-label, randomized controlled trial demonstrated a statistically nonsignificant but clinically significant number of patients with recurrent CVT in the dabigatran group compared with the warfarin group, and a comparable number of clinical major bleeding among the two groups. Ongoing studies, such as the SECRET trial, aim to provide more robust evidence [139] (Table 1). In conclusion, DOACs offer a promising alternative to traditional VKAs in the management of CVT, with comparable efficacy and safety profiles. Further large-scale prospective studies are warranted to confirm these findings and to establish definitive treatment guidelines.

**Table 1:** Summary of evidence on DOACs use in various thrombotic conditions.

Clinical Condition	DOACs	Evidence Strength	Limitations
Chronic Liver Disease	Apixaban, Rivaroxaban, Edoxaban	Moderate [11]	Limited data in Child–Pugh B, contraindicated in Child–Pugh C
Advanced Chronic Kidney Disease	Apixaban, Rivaroxaban (cautious use)	Moderate [40,45]	Limited RCTs for stage IV-V CKD and dialysis patients
Nephrotic Syndrome	Apixaban, Rivaroxaban	Low [55,56]	Limited pharmacokinetic studies
Cancer-Associated Thrombosis	Apixaban, Edoxaban, Rivaroxaban	High [63,74,79]	Increased bleeding risk in GI cancers
Obesity	Apixaban, Rivaroxaban	High [89,93]	Caution in BMI > 50 kg/m^2^
Antiphospholipid Syndrome	Rivaroxaban (not recommended), Apixaban	Low [101,102,103,104]	Contraindicated in triple-positive APS
Embolic Stroke of Undetermined Source	Rivaroxaban, Dabigatran, Apixaban	Mixed [105,107,109,110]	No significant benefit over aspirin
Superficial Venous Thrombosis	Rivaroxaban, Apixaban	Low [114,115,116]	Not first-line; fondaparinux preferred
Upper Extremity Deep Vein Thrombosis	Apixaban, Rivaroxaban	Low [124,125,126,127]	Limited RCTs
Inferior Vena Cava Thrombosis	Apixaban, Rivaroxaban	Low [132,133,134,135]	Limited real-world data
Pelvic Vein Thrombosis	Rivaroxaban, Apixaban	Low [138]	Extrapolated data from other VTE studies
Cerebral Venous Thrombosis	Dabigatran, Rivaroxaban	Moderate [138,139]	Need for larger RCTs

### 3.13. DOACs in COVID-19

The COVID-19 pandemic, caused by the novel coronavirus SARS-CoV-2, emerged in late 2019 in Wuhan, China, and quickly spread globally, leading the World Health Organization (WHO) to declare it a Public Health Emergency of International Concern in January 2020 and a pandemic by March 2020.

COVID-19 presents with a range of symptoms, from asymptomatic or mild illness (fever, cough, fatigue, anosmia) to severe respiratory failure, thrombotic complications, and multi-organ dysfunction. Since the onset of the COVID-19 pandemic, thromboembolic complications have been recognized as a major cause of morbidity and mortality, particularly in hospitalized and critically ill patients [140]. The hyperinflammatory response, endothelial dysfunction, and immobilization contribute to a markedly increased risk of VTE, including DVT and PE [141]. Specifically, COVID-19-related coagulopathy involves a complex interplay of inflammation, platelet activation, endothelial injury, and stasis. Elevated D-dimer levels, fibrinogen, and pro-inflammatory cytokines are commonly observed [142,143]. These changes underlie the increased risk of both macro- and microvascular thromboses, necessitating early anticoagulant strategies.

Several studies have assessed the utility of DOACs in non-hospitalized COVID-19 patients. The ACTIV-4B Trial is a randomized controlled trial evaluating apixaban (2.5 mg or 5 mg BID) versus placebo in outpatients with COVID-19 [144]. The trial was stopped early due to low event rates and no significant benefit in preventing clinical deterioration or thromboembolic events. Currently, guidelines do not recommend routine DOAC use for thromboprophylaxis in outpatients with mild COVID-19. However, patients already on DOACs for pre-existing conditions (e.g., atrial fibrillation, prior VTE) may safely continue therapy.

Anticoagulation is standard care in hospitalized COVID-19 patients, but parenteral agents (e.g., low molecular weight heparin [LMWH]) are preferred due to rapid onset and offset, better control in critically ill patients, potential anti-inflammatory effects, and fewer drug–drug interactions. There is limited data supporting the use of DOACs in the acute hospital setting, especially among ICU patients. The main concerns include fluctuating renal/hepatic function, gastrointestinal absorption issues, and interactions with antivirals or corticosteroids.

Prolonged thrombotic risk post-hospitalization has prompted investigation into extended prophylaxis. The MICHELLE Trial, a multicenter randomized controlled trial, showed that rivaroxaban 10 mg daily for 35 days post-discharge reduced the risk of symptomatic and asymptomatic thromboembolic events in high-risk COVID-19 patients, without a significant increase in bleeding [145]. These results support selective use of low-dose DOACs post-discharge, especially in patients with high thrombotic risk (e.g., elevated D-dimer, limited mobility, comorbidities) and low bleeding risk.

Patients already on DOACs for other indications, such as non-valvular atrial fibrillation and prior VTE, can generally continue treatment unless hospitalized or critically ill. In such cases, switching to LMWH is often preferred for better safety and adjustability.

Drug interactions (e.g., with dexamethasone, antivirals like ritonavir) should be monitored, as they can affect DOAC metabolism, especially those processed by CYP3A4 and P-glycoprotein. Ongoing trials (e.g., PREVENT-HD, XACT) are exploring the role of DOACs in broader outpatient and post-acute COVID-19 populations. 

Practical considerations include the evaluation of renal and hepatic function, especially in severe disease, as well as the assessment of bleeding risk vs. thrombotic risk to guide decisions on initiation, continuation, or switching [146]. Finally, patient compliance and access to monitoring and follow-up are essential in outpatient settings.

In conclusion, DOACs are not routinely used in acute COVID-19 management but have a potential role in post-discharge thromboprophylaxis and in patients with pre-existing indications. Ongoing research will further clarify their role in selected COVID-19 populations. Clinical judgment, risk stratification, and evolving evidence should guide DOAC use across the spectrum of COVID-19 care.

## 4. Conclusions

DOACs have revolutionized anticoagulation therapy, offering a more convenient and predictable alternative to VKAs across various clinical conditions. Their efficacy and safety have been well established in VTE prevention, AF-related stroke prevention, and CAT. However, their use in specific high-risk populations, including patients with chronic liver disease, chronic kidney disease, antiphospholipid syndrome, obesity, and superficial venous thrombosis, remains an area of ongoing research.

Current guidelines continue to evolve, reflecting the need for further large-scale RCTs and real-world studies to optimize DOAC selection, dosing strategies, and long-term outcomes in these challenging populations.

In conclusion, while DOACs are transforming anticoagulation therapy across a range of conditions, their use must be tailored to individual patient risk factors, clinical presentations, and available evidence. Continued research and updated guideline recommendations will be essential to further refine their role in complex patient populations, ensuring both efficacy and safety in real-world clinical practice.

## Data Availability

Not applicable.
